# Free Associations Mirroring Self- and World-Related Concepts: Implications for Personal Construct Theory, Psycholinguistics and Philosophical Psychology

**DOI:** 10.3389/fpsyg.2016.00981

**Published:** 2016-06-29

**Authors:** Martin Kuška, Radek Trnka, Aleš A. Kuběna, Jiří Růžička

**Affiliations:** ^1^Science and Research Department, Prague College of Psychosocial StudiesPraha, Czech Republic; ^2^Faculty of Humanities, Charles University in PraguePraha, Czech Republic; ^3^Department of Econometry, Institute of Information Theory and Automation of the Czech Academy of SciencesPraha, Czech Republic

**Keywords:** personal construct theory, free association, world, self, psycholinguistics, philosophical psychology

## Abstract

People construe reality by using words as basic units of meaningful categorization. The present theory-driven study applied the method of a free association task to explore how people express the concepts of the *world* and the *self* in words. The respondents were asked to recall any five words relating with the word *world*. Afterward they were asked to recall any five words relating with the word *self*. The method of free association provided the respondents with absolute freedom to choose any words they wanted. Such free recall task is suggested as being a relatively direct approach to the respondents’ self- and world-related conceptual categories, without enormous rational processing. The results provide us, first, with associative ranges for constructs of the *world* and the *self*, where some associative dimensions are defined by semantic polarities in the meanings of peripheral categories (e.g., Nature vs. Culture). Second, our analysis showed that some groups of verbal categories that were associated with the words *world* and *self* are central, while others are peripheral with respect to the central position. Third, the analysis of category networks revealed that some categories play the role of a transmitter, mediating the pathway between other categories in the network.

## Introduction

Theoretical concepts that were developed a few decades ago are often understood rather as theoretical foundation stones and may be even seen as outdated in some cases. Sometimes, however, situations occur when some older theory is still very influential and inspiring for the field. In psychology, this is the case with Kelly’s (1955) personal construct theory (PCT), to which the attention of researchers is turned rather frequently. For instance, Home et al. (2010) point out that PCT provides “the theoretical framework to learn which constructs the respondents themselves use, but have possibly not yet articulated, and which avoids introducing constructs that stem from the researcher” (Home et al., 2010, p. 503); Cridland et al. (2014) conclude in their research article on adolescents with high-functioning autism (HFA) that “PCT provides an eloquent and in-depth account of developmental issues for adolescents with HFA” and “propose PCT as efficacious in doing justice to the complexity of this condition during the particular challenging period of adolescence” (Cridland et al., 2014, p. 116); Horley and McPhail (2009) interpret terrorism from the PCT perspective and name PCT as a “well-established if rather unconventional psychological theory” (Horley and McPhail, 2009, p. 119), which enables understanding an issue through the use of construct systems. Recently, there have been apparent efforts among several authors to update and utilize the timeless thoughts of Kelly and his followers, Bannister and Mair (e.g., Bannister and Mair, 1968) or Fransella (e.g., Fransella, 2005).

In dealing with the meanings of words, more precisely: in measuring the meanings of words, the term semantic space is widely employed and two basic ways of delimiting it are distinguished: (1) the traditional one, which is word-based and focused on the co-occurrence of words (e.g., Lowe, 2006), and (2) the syntax-based, which reflects the mutual relations between words (e.g., Geeraerts, 2010). Kelly’s PCT is focused primarily on a person who is actively engaged in giving meaning to the *world* and the *self*. After many years Kelly’s contribution is still considered to be radical, because his personal constructs psychology means abandoning the mechanistic and reductionist traditions in psychological thinking, and it fits comfortably into more recent developments aiming to see man in a holistic perspective (Winter, 2012).

The goal of the current paper is to explore how people perceive and understand the *world* and the *self*, or, more specifically, how they associate these two given terms employed as cue words in a free association task. The process of associating is comprehended as verbal constructing. As Kelly states, “there are always some alternative constructions available to choose among in dealing with the world” (Kelly, 1991, p. 11). Associations of the terms *world* and *self* acquired in the present study are considered to be basic meaning elements of what Kelly calls constructs: “man creates his own ways of seeing the world in which he lives … man might be seen as an incipient scientist … each individual man formulates in his own way constructs through which he views the world of events” (Kelly, 1991, p. 9). Kelly also highlights the importance of how people construct and understand the self: “The self is, when considered in the appropriate context, a proper concept or construct. It refers to a group of events, which are alike in a certain way and, in that same way, necessarily different from other events. The way in which the events are alike is the self. That also makes the self an individual, differentiated from other individuals” (Kelly, 1991, p. 91). Categorizing and further analysis of associations of the words *world* and *self*, both of which are fundamental terms, became the key to our study, inspired by Kelly’s PCT.

## Personal Construct Theory

If we wish to think about what brought us to remove the dust from Kelly’s thoughts six decades after their first publication, let’s begin with a quotation: “We start with a person. Organisms, lower animals, and societies can wait. We are talking about someone we know, or would like to know—such as you, or me. More particularly, we are talking about that person as an event—the processes that express his personality” (Kelly, 2003, p. 7). These words open a perspective in which the individual is in the center. But Kelly gets even closer – he is more personal or intimate. Moreover, he turns this perspective inside out and describes a man as his own “scientist.” And this is not only a particular man as the scientist – “The aspirations of the scientist are essentially the aspirations of all men” (Kelly, 1991, p. 30). If we believe this claim that Kelly makes, the respondents in the present study, when writing their associations, gain the possibility of approaching themselves in this manner. “Thus, just as the experimental scientist designs his experiments around rival hypotheses, so each person designs his daily explorations of life around the rival hypotheses which are suggested by the contrasts in his construction system” (Kelly, 1991, p. 90). The associations gathered in the present study are considered to be the basic elements of these constructs.

Kelly named the core of PCT as the “Fundamental Postulate: a person’s processes are psychologically channelized by the ways in which he anticipates events” (Kelly, 1991, p. 32). Kelly states this postulate not as a dogmatic idea, but rather as a thought-provoking one. “The new outlook which a person gains from experience is itself an event; and, being an event in his life, it needs to be construed by him if he is to make any sense out of it” (Kelly, 1991, p. 55). To associate terms such as *world* and *self* means to come out from individual experience, and, based on that experience and through the verbal categories, to show how the *world* and the *self* may be constructed. Kelly explains the terms channelized and anticipating events as depicting the dynamism of human processes and as cutting off from former psychological stimulus-response determinism (Kelly, 1991).

Kelly himself points out that PCT is more a metatheory than a theory. Ten years after the first publication of his book (Kelly, 1955) he also summarized how PCT was categorized among other authors: as cognitive, existential, emotional, learning, psychoanalytic, behaviouristic, pragmatic, reflective and many other theories, including “no theory at all. It has also been classified as nonsense, which indeed, by its own admission, it will likely some day turn out to be” (Kelly, 2003, p. 8). A period critical evaluation of PCT in a submission by Kelly’s direct followers, Bannister and Mair, was published by Eysenck (1968) in Nature. Eysenck’s (1968, p. 99) review summarizes the imperfections of PCT, namely, that some significant claims of PCT were “achieved purely on a verbal plane.” On one hand, the lack of empirical evidence, which is criticized as an imperfection, is understandable. On the other hand, results based strictly on quantitative empirical evidence can generate conclusions such as, e.g., “the more psychotherapy, the smaller the recovery rate” (Eysenck, 1952, p. 322), which is Eysenck’s famous conclusion from his research on the issue of (in-) effectiveness of psychotherapy, published in Eysenck (1952). Such an argument implicitly contains a question about whether philosophizing in the area of psychology should be practiced at all. In addition, Kelly explicitly delimits his position as philosophical and calls it constructive alternativism. “The best we can ever do is project our anticipations with frank uncertainty and observe the outcomes in terms in which we have a bit more confidence” (Kelly, 2003, p. 5). This statement also explains why the task of free associations was employed as a data-mining method in the present study.

## Methodology

### Subjects

Data were collected from university students, university graduates and young adults of similar age. The majority of respondents were young people, often college students (68%, age between 18 and 33), educated mostly in the humanities (41%), technical or economic fields (29%), and natural science or medicine (9.6%). The final sample consisted of 251 respondents (156 women and 95 men); the median age was 26 years old. Respondents were recruited from Czech universities of different types and from random data collection in Prague, the capital of the Czech Republic. Participation in the study was fully voluntary with no incentives provided for participation. All respondents were Czech native speakers. The research design was approved by the institutional ethics committee. All respondents signed an informed written consent with their participation in the study.

### Materials and Procedure

A free association task (Nelson et al., 2004) with multiple associations to the cue words was used in the present study. The main aim was to explore the conceptual categories that respondents retrieve when they are asked to recall associations to the word *world* and the word *self*. In the free association task, the respondents are asked to write the first word that comes to mind that is related or strongly associated to the presented cue word. The method of free association provides the respondents with absolute freedom to choose any words they wanted. Such free recall is suggested as being a relative direct approach to the respondents’ self- and world-related conceptual constructions.

At the beginning, respondents filled in basic demographical characteristics. Then the cue words were presented to respondents on a sheet of paper. Two cue words were used: the word *world* and the word *self*. The respondents were asked to recall any five words relating with the Czech word for *world* with the instruction “Please, write five words that you recall when you hear the word *world* to the following five lines.” Five short horizontal lines placed below each other were offered to respondents below the instruction. Afterward respondents were asked to recall any five words relating with the Czech word for *self* with the same instruction and response design. The time for writing the associations was not limited.

The present research went the opposite way when compared with the method of controlled word association test (e.g., Woodworth, 1921) in that we did not apply predefined categories from which the respondents had to choose particular words. The aim of the present study was to categorize all of the acquired associations by their prevailing meanings into groups in order to subsequently display the distribution of categories and inter-category relations.

In the sense of delimiting the semantic space, the method of the present study is word-based, and because we work with particular associations which were not articulated in form of sentences or narrations, we also visualize the relations between words other than the grammatical. Jackson and Bolger (2014, p. 12) suggest in their recent article about a high-dimensional graph of semantic space that “perhaps the method of calculating relationship, rather than representing relationships, is what differs between relationship types.” We suggest trying to avoid the use of the term “semantic space” in the present study, with respect to the employed method of coding, which reflects only one prevailing meaning of an association in the given context. In this way, each categorized association represents a one-directional vector, which creates no space *per se*. This position seems to be quite radical, especially in the context of contemporary computational psycholinguistics, where the number of dimensions of semantic space has no limit and is usually calculated with multi- or high-dimensionality. Also, the authors of this article would otherwise prefer a multidimensional space construction, if appropriate, as was used in their recent article dedicated to emotional space (Trnka et al., 2016). But here, instead of dealing with the semantic space of particular words or collocations, we (1) describe and explain the meaning of each category of associations and (2) reconstruct the distribution of categories in two dimensions, separately for associations of *world* and *self*.

### Data Analysis

Acquired data in form of individual words or short phrases were analyzed separately for the cue words *world* and *self*. Firstly, the data were sorted by frequency of occurrence of particular words. No data pooling was employed, so singulars and plurals, various verb tenses etc. remain intact as gathered. The most frequent words with particular meanings were suggested as category names. Secondly, data were categorized by the meaning of each association. For associations of both cue words, *world* and *self*, two separate categorization systems were developed. The exhaustive categorization process left no associations uncategorized. All categories were derived from an examination of the data (e.g., Aylwin, 1977). In the case, when a word can be assigned to more than one category, the prevailing meaning of the given word was employed. An independent rating scored an almost 82% match in the ex-post sorting of the associations into categories, namely 82.4% for *world* associations (κ = 0.913) and 81.6% for *self* associations (κ = 0.909), both at *p* < 0.001 level.

For both cue words, *world* and *self*, we elaborated two 251 × 18 incidence matrices. In each matrix related to the mentioned cue words, rows represented respondents and columns represented particular categories. The cell gained the value *a_nk_* = 1 when the respondent *n* reported at least one association of the category *k*, and the value *a*_nk_ = 0, when the respondent reported no association. Incidental matrices act as an entry for two types of analysis borrowed from psychometrics and multivariate statistics. These two methods are designated for mapping statistical connections of the mutual occurrence of particular categories. The first method is factor analysis (PCA) (Kline, 2013), and the second is the technique of Inverse covariance matrix (Whittaker, 1990). The output of factor analysis (PCA) is the mapping of each item to the smaller Euclidean space (Überla, 1968), which is in psychometrics significantly less dimensional due to correlation proximity. Coordinates in this space allow each particular item to be described as the vector of several factors with sufficient individual exactness maintained.

The goal of an inverse covariance matrix is to distinguish direct statistical dependencies of particular items (categories) from transmitted, indirect correlations. The output is a network of direct statistical dependencies on the level of a statistically typical respondent, which allows a pathway (or more pathways) of inter-category dependencies to be reconstructed between more indirectly dependent categories. The number of factors in the factor analysis was defined using the technique of point of curve-break of descending eigenvalues. For both cue-words, the corresponding break-value of the eigenvalue was 1.5 – the factor was accepted when it explained at least 50% more variance than the mean value allocated to the particular category. For both of the cue words, *world* and *self*, this allowed a given items to be mapped by two factors. Varimax rotation was used for the resulting two-dimensional maps.

## Results

### Associations and Category Frequencies

Two discrete categorization systems were developed, one for associations of the cue word *world*, second for associations of the cue word *self*. Both systems consist of 18 categories (see **Tables 1** and **2**).

**Table 1:** Description of categories of *world* associations.

Nature	Animals, plants, animate and inanimate nature. Nature’s elements. Natural phenomena and processes. Dimension: all on the planet Earth (but the Planet as a whole belongs to the Space category).	Life, air, forest, wind, sand, elements, trees, light, continents, oil, birth, cloud, sky…

Aqua	Water. Discrete subcategory of Nature. Everything on water and about water.	Water, sea, ocean, oceans, waterfall, underwater, river…

Big	Great. Discrete subcategory of Nature, pointing out the largeness.	Largeness, big, expanse, scope, endless, infinity…

Space	Universe. Planet Earth as a whole, its characteristics. Cosmos. Widest possible scope.	Space, planet, Earth, globe, rounded, Sun, everything, sphere, equatorial…

People	Human (culture is separated in the next category).	People, population, human, man, mankind, inhabitants…

Culture	Culture according to its anthropological definition, which means, in the widest extension. i.e., as the product of human behavior, material and non-material, including values, systems, achievements and products. Including processes, situations, symbolic systems.	Culture, art, politics, money, cities, music, communication, God, society, technology, globalization…

Positive	Positive values in general, positive associations.	Beauty, nice, fun, joy, peace, love, happiness, party, harmony…

Challenge	The world as the challenge.	Challenge, adventure possibilities, discovering, curiosity, interesting…

Negative	Negative values in general, negative associations.	Horrible, pain, evil, cruel, dirty, destruction, madhouse, crisis…

Trouble	Serious problems of the world. Discrete subcategory of Negative.	War, catastrophes poverty, violence, corruption, injustice hunger, threatened…

Travel	Traveling. Evaluation (positive and negative) does not belong here.	Journey, travel, holiday, maps, vacation, Phileas Fogg, navigation, traveling…

Diversity	Variety. If associations reflect differences and diversity, they belong here. Possible overlapping with Culture to be resolved by putting of plurals here, if they refer to diversity (e.g., cultures, languages etc.).	Atlas, nations, other cultures, diverse, complexity, heterogeneity…

Social bonds	Social relationships (and their background).	Relationships, family, friends, parents, children, friendship, grandparents…

Color	A particular color.	Colors, blue, green, gray, deep blue…

Linguistic	A special association within the Czech language (e.g., a rhyme, lexical basis contains the Czech word for world, etc.). Specific Czech facts.	(Not translatable)

Wow!	An expression of amazement, impression. Needs to be balanced with Big, where largeness is the matter (but not astonishment from the largeness).	Miracle, uniqueness, eternity, huge, unbelievable, surprising, magic…

Together	Jointly. The expression of mutuality. “We all live in the one world.”	Togetherness, we, brotherhood, integrity, wholeness coexistence…

iWorld	Me-world, appropriation of the world, I am the world.	My, my life, me, my playground, I, is mine, this around me…


Both of these descriptions (in the Czech language) have also been provided to an independent rater for the ex-post sorting of the associations into categories.

The word *world* was associated most strongly to words from categories which relate to nature’s essence of the world in a local, global or cosmic dimension. Special meaning was dedicated to the element water. Civilization and its achievements – or an anthropocentric paradigm – were sorted in more less extensive categories, with respect to different meanings of associations.

The word *self* strongly reflected individualism, including personal settings and various states of being. All associations were sorted distinctively in particular categories by their meanings, as **Table 2** displays more in detail.

**Table 2:** Description of categories of *self* associations.

Trait	Personal attribute, characteristic. Positive and negative self-evaluation, marking of self with a particular characteristic or ability. A particular personality trait or character.	Responsibility, effort, reliability, creativity, intellect, selfish, asshole, slacker…

Value	Worth, but because not evaluating, also their opposites. Note: the difference from Trait is often only in that Trait is the accusative form and Value the nominative form. Beware, that Value can be also accusative, if not expressing a Trait directly (example: equal = Value, but not Trait).	Freedom, understanding, health, calm, space, honor, tradition…

Individual	I as the initiator (performer), originator and center. Self-highlighting (also in a negative way). This is not a Trait or Value!	Me, ego, human, person, creature, personality, ambition, center, king, individuality…

Embodiment	Everything concerning the body. Physical. Evaluation of the body, esthetical and in time. Body activities and abilities. Body reactions. Body language (non-verbal communication).	Smile, beauty, power, old, cry, laugh, brain, skeleton, weight…

Existence	Being and consciousness of it, experiencing of it. Questions on being. Trajectory and direction of being. Dimensions of being. My being. Doubts concerning being. Terminality of being.	Life, transience, be, terminality, nothing, who?, paradox, journey, soul, fight…

Social	Society. Social bonds of all kinds, including experiencing their absence.	Friendship, relationships, friend, he, she, we, partner, people, together, loneliness, buddy…

Family	A distinct subcategory of Social, including types of family bonds, and offspring.	Mother, parents, wife, children, sister, brother, son, daughter, family…

Gender	A distinct subcategory of Social. Only the indication of gender, without the possibility of recognizing any social bonds.	Woman, man, girl, gay, boy…

You	A distinct subcategory of Social. Separate group of associations, where “I” associates with “you.”	You, you and me, what about you?…

Name	Own names or names of someone else, incl. Zodiac signs.	(Different names), Brad Pitt, Freud, Èapek, my name, Asterix, Capricorn…

Emotion	And feelings concerning the self. Expressions of like and dislike, Emotionality and its experience. Experienced emotions.	Joy, happiness, fear, sadness, stress, desperation, compassion…

Love	A distinct subcategory of Emotion. Everything, where love is explicitly occurring. Including negation of love and its manifestation.	Love, like, flirt, sex, tenderness, I love you, adored, loving…

Leisure	Leisure time activities. That which prevailingly associates with free time. That is, I and “something” (a given association) predominantly represent my free time.	Sport, dance, music, football, beer, travel, run, weekend, singing, art, book…

Professional	Profession, occupation, job. That which concerns work and preparation for a profession. Professional branches and domains.	Work, student, employment, factory, maid, dentist, sociology, school…

Material	Material background, things, tools. Material equipment. Consummated things. Materialism and related point of view on reality, incl. other people.	Home, money, car, household, shopping, train, treasure, welfare, bed, food…

Nature	Animals, plants, inanimate nature. Natural elements and resources. Natural phenomena.	Sea, bird, nature, dog, parrot, coast, nests, country, sun, fire, mountains, miaow…

Fun	Joy, amusement. “I” is associated with cheerfulness of all kinds.	Humor, fun, giggle, funny, hilarious, amusement, funny…

Crisis	Crisis and emergency situations. Mess. Border situations. Consternation or anxieties from the self.	Disaster, illness, chaos, crisis, collision, fall, worry, overpressure…


**FIGURE 1:**
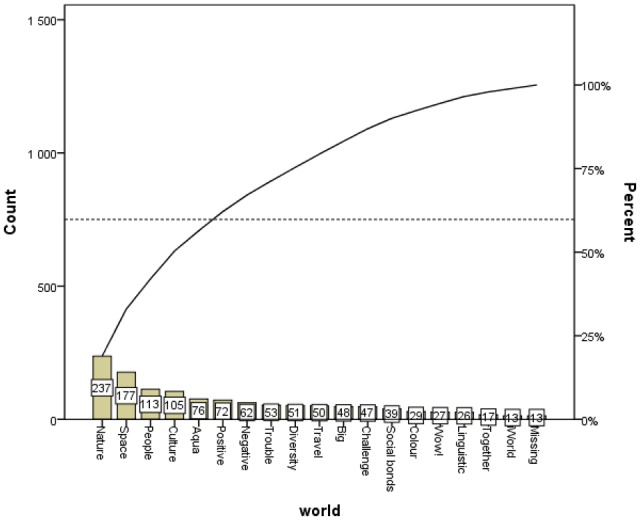
**Overall association distribution in categories**.

**FIGURE 2:**
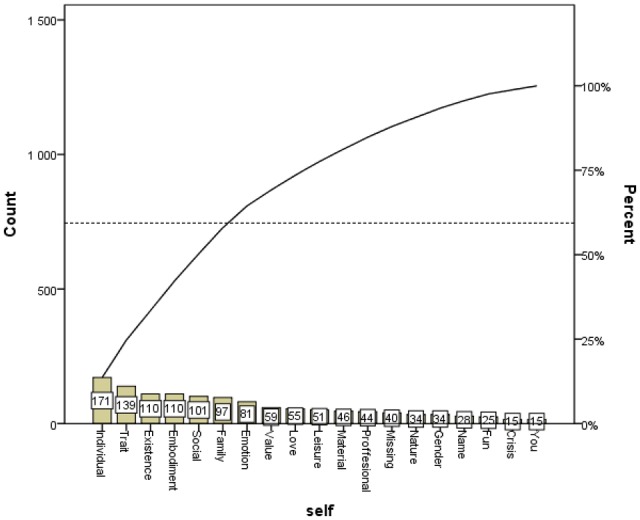
**Overall association distribution in categories**.

The Pareto chart shows on one side the frequencies of the most commonly appearing categories, while on the other side it shows that after the initial most common categories, which for the cue word *self* are Individual, Trait, Existence, Embodiment and Social, the level of 60% was achieved within the named categories. It also shows that other categories, starting with Family, appeared rather rarely (see **Figures 1** and **2**).

### PCA Analysis

To display the various ranges of how people associate the world and themselves, PCA data analyses were conducted, separately for the cues *world* and *self*.

**FIGURE 3:**
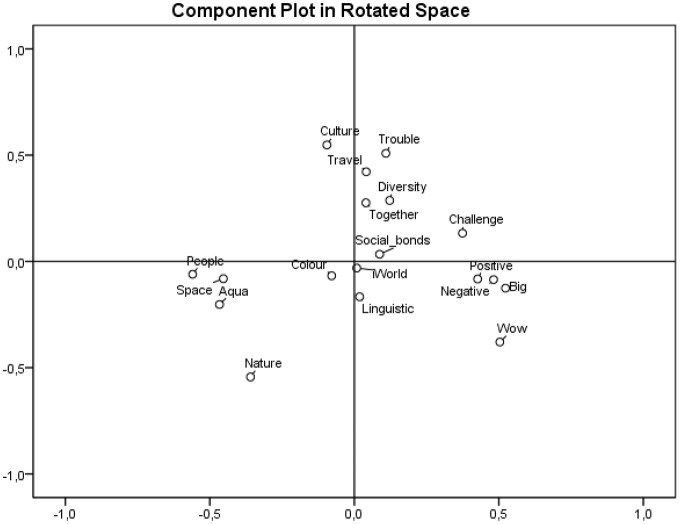
**Component plot in rotated space chart for *world***.

The component plot in a rotated space chart for *world* associations (**Figure 3**) shows the distribution of categories in two basic dimensions (ranges). The iWorld category in the central position represents the point of origin of vertical and horizontal axes, which correspond to following ranges: a vertical axis with polarities Nature vs. Culture and a horizontal axis, where the range is triangulated by concrete categories (People, Space, Aqua) on the left side and by abstract categories (Challenge, Positive, Negative, Big and Wow!) on the right side.

The vertical range demarcated by the categories Nature on one side and Culture on the other side shows the two opposite directions of how people associate the *world*. The central position of the iWorld category corresponds very well to the basic postulates of PCT, where the individual is the key determinant of how people reflect the *world* and the *self*. Although the iWorld category occurred only rarely, its position marks the center about which personal constructs for the *world* oscillated.

**FIGURE 4:**
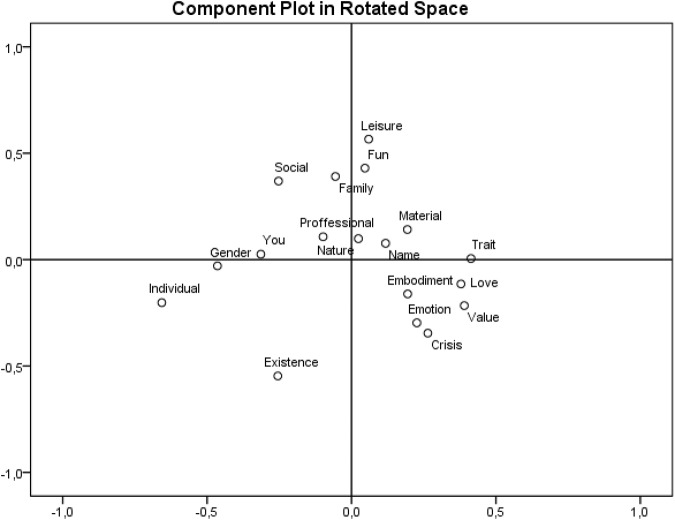
**Component plot in rotated space chart for *self***.

The component plot in a rotated space chart for *self* associations (**Figure 4**) displays the distribution of categories into four groups: (1) categories like Leisure, Fun, Family and Social grouped in the upper part of the data projection are examples representing some aspects of everyday life; (2) the categories Trait, Love, Embodiment, Emotion, Value and Crisis are related with personality structure; (3) alongside, the categories Value, Crisis and Existence in the bottom part of data projection represent the philosophical context of being; and (4) the left part of the chart groups the distinctly personal categories: Individual, Gender and You.

### Category Networks

The inverse covariance matrix express the system of statements ρ_A,BjX_∼0, where A, B are categories, and X is any kind of knots which separate vertices A and B in the network. ρ_A,BjX_ is coefficient of partial correlation of articulation of A and articulation of B by random subject, controlled for set X. Category network approximates real dependence structures, which are generated from the standardized inverse covariance matrix.

**FIGURE 5:**
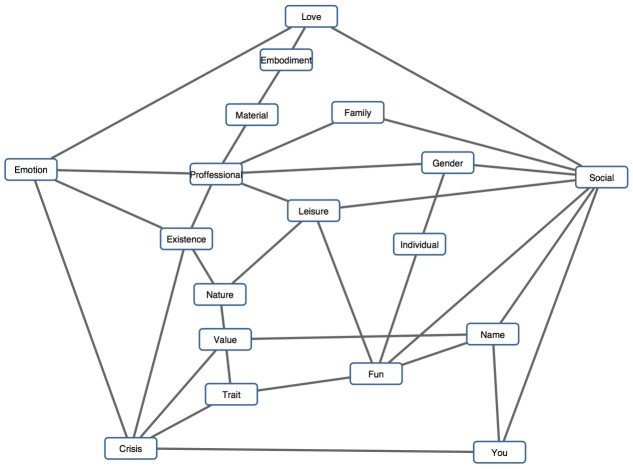
**Category network for *world***.

**FIGURE 6:**
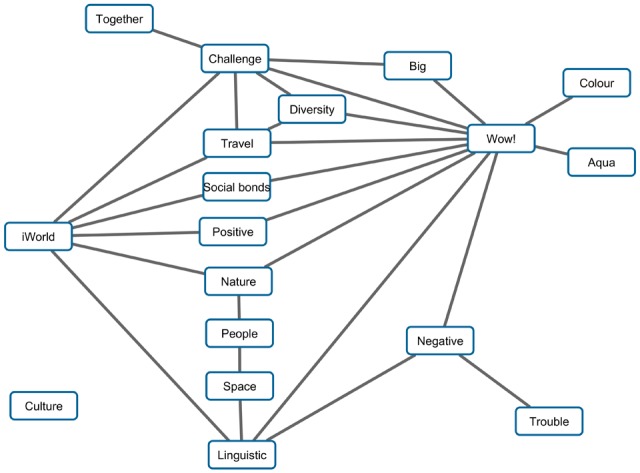
**Category network for *self***.

On the basis of the inverse covariance matrix, which analyzes data on the level of individual respondents, two networks of categories were modeled, one for the categories of the *world* associations, second for the associations of *self*. The individual is the numerator of the links. The purpose of the inverse covariance matrix method is to distinguish two types of dependencies, direct and mediated dependencies. An edge connects the nodes directly. If another category is placed between two categories, such a category acts as a transmitter, which mediates a pathway between them and illustrates the mediated dependencies (see **Figures 5** and **6**).

Lines display the direct not-mediated probability dependence. The probability of a common occurrence is independent of other category constellations. As an example, let’s take a look at the Linguistic and Trouble categories: if they occur together in the same respondent, the category Negative acts as the mediator here. The only lone-standing category is Culture. This means that Culture, even if this is the fourth most commonly occurring category, has no probability dependence to be found with another specific category more or less as with others. Such a category does not elicit any category more than any other.

## Discussion

The present theory-driven study provides new empirical evidence that contributes significantly to the development of PCT. Aside from this; our results also have implications for other fields, such as psycholinguistics, associative priming, or philosophical psychology. These implications will be presented within the particular subsections, but at the beginning we will briefly summarize the main findings of the present study. First, the results provide us with the associative ranges for the constructs of the *world* and the *self*. The results showed that some associative dimensions are defined by semantic polarities (e.g., Nature vs. Culture, everyday life vs. philosophical context of being). Second, our analysis showed that some groups of verbal categories associated with the words *world* and *self* are central, while others are peripheral in regard to the central position (see the results of PCA analysis in **Figures 3** and **4**). Third, the analysis of category networks revealed that some categories play the role of a transmitter that mediates a pathway between two other categories in the network (see **Figures 5** and **6**). Interestingly, one category, named “Culture,” was determined to be an independent category in the analysis of category networks, probably without any prevailing dependence with another category. All of these findings help us to better understand the way that our respondents constructed verbal associations with the constructs of the *world* and the *self*, but also the mechanism of personal construction in general.

### Implications for Personal Construct Theory

Kelly’s PCT is on one hand considered as essentially important; on the other hand, the lack of empirical evidence supporting his theory has complicated its broader impact. We still consider Kelly’s PCT as a not very empirically verified reservoir of inspiration, which calls for rediscovering. We assume, in line with our results, that such rediscovering, especially when conducted with the use of empirical methods, will broaden PCT and the possibilities of its implications. Even after half a century, some scholars (e.g., Butler, 2006) are pioneering in the gathering of empirical material which is employed to specify the content of Kelly’s theoretical assumptions. Like Butler (2006), who is empirically searching for the content of Kelly’s core constructs, the present study aims to provide new insight on the relations between meaningfully categorized words originating from a free association task. The present study used a dynamic way of categorization, which allowed us to work with each arbitrary association, expressed as a word or collocation. Subsequent statistical operations enabled the data to be visualized, separately for associations of *self* and *world*, and opened a space for interpretation of the displayed distributions and relations. Although the present study is not working with a large language corpus, as many recent studies have (e.g., Nelson et al., 2004; Jackson and Bolger, 2014), our data enabled the significant relations between categorized associations to be visualized.

The results of our study led us to reconsider three of the eleven ground stones on which Kelly elaborated the fundamental postulate (see the section PCT) of his PCT. Firstly, we broaden the basic concept of the dichotomous foundations of the construct that Kelly stated and named as a dichotomy corollary; secondly, we reformulate his definition of the choice corollary; and, thirdly, we suggest reducing the number of ground stones of PCT to 10, via complete removal of the range corollary.

Kelly’s proposition, which he named the dichotomy corollary, states that people construct their reality on two opposite alternatives. This point was strongly criticized, e.g., by Riemann (1990). Given the results of the PCA analyses conducted within the present study (see **Figures 3** and **4**), we are able to identify more than only opposite alternatives; moreover, we can reconstruct the entire space where *self* and *world* are emerging as constructs and which are not necessarily based on contrasts (see the section PCA analysis). In this manner, we extend Kelly’s original statements.

We assume that Kelly partially anticipates the fact that the foundations of constructs which are not dichotomous also exist. On one hand, he argues that “A construct is the basic contrast between two groups” (Kelly, 2003, p. 10), and on the other, he operates with a “finite number of dichotomous constructs” (ibid.). In other words, the number of dichotomous constructs is limited, which can elicit the question of whether there are also other, non-dichotomous constructs. We guess that here is a hidden space for formulating the fundaments of constructs other than dichotomous ones, the space which is captured as result of PCA analysis (**Figures 3** and **4**).

Further, the need for the upgrade of another ground stone, the choice corollary, stems from previous reformulation of the dichotomy corollary, which is considered to be the most important such ground stone. The main contribution of this update is the broadening of the space where alternatives other than the dichotomized can be considered. The distributions of categories, as shown by the results of our PCA analyses, as well as the number of categories – 18 for each of the cue words – show a higher complexity of constructs, where groups of categories were identified, including their central or peripheral positions. Regarding the above-mentioned, we suggest removing the word *dichotomized* from the definition of the choice corollary. The definition will thus read: A person chooses for himself the alternative in a construct through which he anticipates the greater possibility for the elaboration of his system (see Kelly, 2003, p. 11).

Last, but not least, the range corollary, which deals with the issue of a limited number of dichotomous constructs, is groundless in the light of reformulating the previous two fundaments of the PCT. Kelly stated that the range corollary as “A construct is convenient for the anticipation of a finite range of events only” (ibid), and he subsequently argues that “Not everything that happens in the world can be projected upon all the dichotomies that make up a person’s outlook. Indeed I doubt that anyone has ever devised a construct that could cover the entire range of events of which he was aware. There are patches of clouds in every man’s sky. This is to say that the geometry of the mind is never a complete system” (ibid). We understand this quoted paragraph also as a collision with how Kelly delimited the borders of his theory. When we set aside the precondition that constructs are based only on dichotomies, as we suggest above, and what the results of present study illustrate (see results of PCA analysis), we can also consider this Kelly’s skepticism as pointless. In this manner, we extend Kelly’s original statements; we even reduce his 11 corollaries to 10.

### Implications for Psycholinguistics

One of the large topics in contemporary psycholinguistics relates to the term *mental lexicon*. The unflagging interest of scholars about this subject has been elicited by Aitchison (1987), who called it a *human word-store*. To explain the term, the following definition can be employed: “A mental lexicon refers to the words that comprise a language, and its structure is defined here by the associative links that bind this vocabulary together. Such links are acquired through the experience and the vast and semi-random nature of this experience ensures that words within this vocabulary are highly interconnected, both directly and indirectly through other words” (Bruza et al., 2009, p. 363). Within such a delimitation, the present study gathered words from a free association task, where associative recall was primed by the cues *word* and *self*. These association chains were interconnected on the individual, personal level (for examples, see the **Tables 3** and **4**).

**Table 3:** *World*: selected association chains (in rows).

Big	Horrible	Cruelty	Injustice	Life
Pain	Ball	Ocean	Life	Death
People	Envy	Nature	Progress	Backstabs
Hazard	Future	Children	Family	Worry
Sea	Courage	Nature	People	Caution
Love	Family	God	Life	Eternity


**Table 4:** *Self*: selected association chains (in rows).

Boy	Jerk	Moron	Cool	Brother
Buffoon	Drunkard	Man	Brad Pitt	Superman
Uncertainty	Fear	Confusion	Mom	Good girl
Killer	Ideas	Lie	Mind	Punishment
Speed	Stress	Time	Work	School
Batty	Emotionally labile	Own world	Helper	Life


Also, subsequent PCA analyses showed relations between categories, where inter-category links were calculated on the basis how individuals articulated their association chains (see the category networks of *word* and *self* in **Figures 5** and **6**). Such data visualization can be considered as an innovative contribution of the present study, because previous analyses in psycholinguistics have been primarily focused on the level of relations between particular words, as in the following example: “Words are so associatively interconnected with each other they meet the qualifications of a *small world* network, wherein it takes only few associative steps to move from any one word to any other in the lexicon” (Bruza et al., 2009, p. 364). In our study, we revealed the mediated dependencies between categories emerging based on the respondents’ associating, and these dependencies created networks (**Figures 5** and **6**) having characteristics similar to the *small world* networks presented by Bruza et al. (2009). Therefore, our study also extends the theoretical assumption of Bruza et al. (2009) for the case of broader semantic spaces, as coherent categories of meaning are suggested. So, according to our analysis, we may rephrase the above-mentioned definition for the level of categories: categories with coherent semantic meanings emerging from human associating are so interconnected with each other that they meet the qualifications of a network of *small world of associations*, wherein it takes only a few steps to move from any one category to any other category. The links shown between the categories in our analysis showed probability dependences, indeed, as explained in the Category network section. Thus, the present study provides innovative insights into the deep structure of the *mental lexicon*, especially for the instance of associate reasoning and subsequent recall of verbal concepts.

When discussing results concerning the semantic meanings of words, we can also turn our attention to classical authorities in the field. One of first sources of inspiration is the work of Osgood et al. (1957) and his semantic differential. The task of measuring semantic differential requires analyzing the given words in opposite dualities; Kelly (1955) also worked with bipolar dimensions of constructs in his PCT. The results of the present study indicate that some of the associative dimensions that emerged from the associations of our respondents also have the character of opposite dualities. The meanings of categories that are located on the vertical and horizontal peripheral sections of the associative range for the *world* are in semantic opposition (**Figure 3**: Nature vs. Culture, concrete vs. abstract categories). On the other hand, the horizontal dimension in the associative range for the self does not show such distribution. One may ask: what does this mean? It is necessary to distinguish two levels of analyzed data: first, the level of associations, and second, the superordinate level of categories. On the first level, it is possible to place associations in the form of accusatives and verbs between two polarities, if it is not a polarity itself. In other words, the position of each such association is definable by its position in the range between corresponding polarities, defined by Kelly as the *extreme points of constructs*. In linguistic categorization, nouns, adjectives and various types of verbs are the basis for sorting words on the scale concreteness–abstractness (e.g., Carnaghi et al., 2008). Because the present study acquired associations of all word classes and because of the aim to categorize all of gathered data by the prevailing meaning of the words or short phrases used, another sorting on the first level wasn’t employed, even though there is a wide scale of methods by which these data could be analyzed (e.g., Nelson et al., 2004). However, new insight on the concreteness–abstractness dichotomy was provided, when PCA analysis of the categories from the term *self* was conducted (see **Figures 3** and **4**).

Our data are not sufficient to conclude that people construct the *world* and the *self* in the manner of opposite dualities, but analysis of the acquired data is able to show that respondents do employ non-opposite mechanisms of semantic construction when associating the given cue words. Nonetheless, it is too early to make such a general conclusion, and the fact that this interpretation is supported only by the results of one empirical study should be taken into account. On the second level of the analyzed data, some very significant opposite dualities are illustrated among the categories by the PCA method. Of course, not all categories in the PCA projection were located in the sense of opposing dualities, which supports another possible way of interpreting of results, as employed by the distribution of the *self* categories (see **Figures 3** and **4**). When analyzing the data on the level of categories (for example Nature and Culture, see **Figure 3**), their distribution in the given example may also be understood as *extreme points of constructs*, if using Kelly’s words.

Further, it is also necessary to stress that the experimental design used in the present study involves the effect of priming. The respondents were provided with the cue words, *world* and *self*, and these cue words influenced their follow-up retrieval of associations from memory. Stacy et al. (2006) pointed out: “Thus, word association seems to measure some sort of relationship that has relevance well beyond the word association task itself” (Stacy et al., 2006, p. 77). Both of the words, *world* and *self*, served as prime words leading to a related target – a chain of word associations in the present study (for examples, see the **Tables 3** and **4**). It is very important to keep in mind this priming effect when exploring the results of the analysis of category networks. This analysis revealed that some categories played the role of a transmitter mediating a pathway between two other categories in the network. Just the presence of transmitters or mediators illustrates very well the sequential nature of our data. Analyzing the problem of sequentiality in chains of words in associative tasks would be an interesting challenge for future studies, but it lies beyond the main scope of the present study, which was focused mainly on the investigation of associative ranges for the constructs of the *world* and the *self* within the theoretical framework of PCT.

### Implications for Philosophies Regarding Self and World

This paper is basically an empirical study, not a philosophical one. Despite this fact, however, some interesting implications for philosophies regarding *self* and *world* can be derived from the acquired data. Generally, we understand the implications introduced below to be a link between the realms of theoretically driven empirical research study on one hand and the field of philosophical thinking on the other. These implications are thought mostly to be preliminary incentives for future developments in the field rather than final statements.

At the beginning, it is important to realize the nature of our experimentally acquired data. Simply put, the respondents recalled their first five associations with the words *world* and *self*. Therefore, we may afford to discuss these associative findings within the context of the phenomenology of Husserl. According to Husserl’s (1970, 1977), our mental processes are essential for the construction of the objective world. Through and by our mental processes we create our own ideas about our existence *in* the world and belongingness *to* the world. From this point, the associations that were gathered from our respondents showed us the underlying structures that constitute the inner meanings of the world’s belongingness. For example, when we look at **Figure 3**, there are many possible concepts of how people may understand their being in the world. The free association task enabled us to approach the inner meanings of the *world* in our respondents. But the question arises: what is the source of these meanings?

Husserls (1970) speaks about *a priori* structures that may be responsible for our belongingness with the world. Categories such as Space, Aqua, Positive, or Negative (see **Figure 3**) are examples of concepts that could even be regarded as some kind of *a priori* structures pre-established in our brains. However, all associations are also primarily subjective. The role of cognition should not be omitted here. Cognition definitely varies between subjects, and free associations are suggested to emerge into the consciousness of a person because of that person’s performance of cognition (*Erkenntnisleistung*) (Husserls, 1977). Cognition is generic within the free associative task, because it generates various associative meanings when faced with stimulus priming. As seen in **Figure 3**, some kinds of categories seem to be less general, e.g., Social bonds, Travel or Linguistic. A person’s performance of cognition (*Erkenntnisleistung*) (Husserls, 1977) plays an important role in the free associative task, and we argue that the gathered associations may be the expression of *a priori* structures but also influenced significantly by a person’s cognition. The concepts that respondents have created in their minds have been constituted (and are still being constituted) in the subject’s constitutional history. And because the associations presented in this study are related to the *world* and the *self*, we may even speculate that these associative concepts can mirror even the core, fundamental concepts of a person’s being in the world.

Perhaps it is too early to make such statements, and we should be careful to come to such a conclusion. Therefore, we instead adopt a less daring position here and satisfy our inquiry with the notion that free association may provide us with signs of the deep mental processing of reality. But there is another interesting implication relating to the mutual inter-relations of associations in our results. As shown by the analysis of category networks (see **Figures 5** and **6**), we may suggest that the concepts are not disconnected from each other in the respondents’ memories. The analysis of category networks revealed a high number of mediated dependencies that are depicted in **Figures 5** and **6.** This informs us about the deep structuring of the inner perception of the world in people’s minds. According to Husserls (1970), all objects are experienced in consciousness, and therefore, all meanings are always constituted in the constitutional history of a subject. Our results showed (see **Figure 5**) that concepts relating to the world are mostly inter-dependent, and that one concept often acts as a transmitter for another concept or concepts. At this point, it would be speculative to consider mediated dependencies between concepts to be related with Husserl’s (1967) basic eidetic laws of passive genesis, but such question may be considered.

Our experimental study worked with verbal stimuli, and respondents also recalled verbal associations to the words *world* and *self*. The choice of this research design is not surprising when working in the field of philosophical psychology. Heidegger (1985) pointed out that our practical encounter with the world around us is encapsulated in our language. In other words, language itself is suggested to mirror our belongingness with the world. Our results (**Figure 4**) showed that self-related associations are grouped into spheres that are related either to some aspects of everyday life (e.g., categories such as Leisure, Fun, Family and Social) or to the philosophical context of being (e.g., categories such as Value, Crisis and Existence, in the bottom part of **Figure 4**). This patterning indicates that Heidegger’s (1985) practical engagement with the world is not restricted to the realm of everyday activities, but also covers engagement with the world that can be considered to be the philosophical context of being of human existence. This existential dimension of being in the world is present in the associations of our respondents, which indicate that the existential aspects of life are also an important part of verbalized Expression (*Aussage*) in Heidegger’s (1985) sense.

Further, it is also possible to posit the broader philosophical question of how many categories are optimal to categorize the world? This question cannot be answered easily, but we may consider some possibilities here. Lowe (2006) posed a similar question in the framework of his four-category ontology, and according to this *squared* way of thinking he concluded, not surprisingly, with four components. In contrast, our category system, which emerged based on empirical evidence, included 18 categories for associations of the word *world* and the same number of categories for associations of the word *self* (see **Tables 1** and **2**). Each category represents a group of verbal associations with similar semantic meanings (see **Tables 1** and **2**), and the frequencies of occurrences are different in different categories (**Figures 1** and **2**). When dealing with continuous attempts to categorize the world from the ancient times up to the present (for a review, see Hackett, 2014), some scholars have even posited the opinion that no categories exist at all. This is the case of the work *The No-Category Ontology* (Bueno et al., 2015), where instead of categories the authors introduce concepts which “can always be revised, refined and recast” (Bueno et al., 2015, p. 233). This fascinating discussion is currently very topical, and we believe that the empirically based results of the present study contribute significantly to it.

Finally, we would like to briefly outline the implication of our study in respect to the position of the concept of *self* in philosophy. Although most psychologists do not have any problems with the concept of *self*, some philosophers have questioned the existence of the *self* in terms of an essential, subjective nucleus of a person. For example, Hofstadter and Dennett (1981) rejected the existence of the *self* and regarded the *self* as a kind of illusion. This approach is very inspiring. Although the words “self” or “I” are included in most of human languages, we may adopt the illusionary nature of the underlying concept of *self* for the interpretation of our results. If we consider the concept of *self* to be an illusionary one, the *self*-related associations that have been gathered from our respondents become much more interesting in this light. If *self* is an illusion, then associations to the word *self* may be helpful in revealing the relationship of this illusionary concept to other related concepts. When looking at **Figure 2**, the word *self* is most frequently associated with concepts falling into the categories “Individual,” “Trait,” “Existence,” “Embodiment,” “Social,” and “Family.” This indicates that people usually understand themselves as individuals with some kind of traits. The self has not been consciously regarded as an illusion, because respondents frequently recalled words relating to the realm of real existence. They associated the *self* with a really existing entity embodied in a particular material form, i.e., a human body. Many associations were also targeted at the social domain of human existence, where *self* was associated with words falling into the categories “Social” and “Family.” We do not aim to use our results for a discussion about the existence or non-existence of the concept of *self* in philosophy. Indeed, we admit that the *self* could be an illusionary concept, and our results (see **Figure 2**) indicated that although illusionary, the *self* was mostly associated with the conceptualization of a socially living individual that has both a material body and psychological traits. It is necessary to say that this embedding of the *self* was found in our population sample, and cross-cultural differences can be expected when conducting the same experiment in another cultural environment.

## Conclusion

The present study reconsiders the PCT in light of the analyses of data acquired in a free association task. The results of this study develop the theoretical concept of Kelly (1955), which has often been criticized for the lack of empirical evidence supporting the PCT. The acquired data were categorized by the dynamic classification method, creating different meaning categories for the cue words *world* and *self*. The categories are based on the acquired data, and the mentioned dynamic disposition of the method simply reflects any data content.

Subsequent analyses illustrate the sphere which was partially outlined by the PCT as dichotomies, but which to our knowledge nevertheless were not shown via the PCA or inverse covariance matrix methods. Categories are distributed in a two-dimensional range, e.g., in central and peripheral positions, semantic polarities and groups. Further, analysis of category networks detect which categories have prevailing dependencies toward other categories when associating *world* and *self*, and which categories are transmitters to other ones. The inverse covariance matrix method also provided an innovative upgrade of the association-to-association links (Nelson et al., 2004) on the level of category-to-category links.

According to the results of the conducted analyses, the present study suggests implications in three areas: (1) in the PCT, we reformulated two of the 11 ground stones which Kelly named as corollaries. After this reformulation PCT is capable to reflect that the constructing of *self* and *world* is not based only on contrasts, as also our analyses depict. Fundaments of constructs different than dichotomous fundaments are also illustrated here. Furthermore, it is suggested that one corollary should be completely removed; (2) in the area of psycholinguistics, the extension of the concept of *small world* network (Bruza et al., 2009) is suggested. Categories emerging from how people associate are linked based on their probability dependences in the present study, which enables an innovative insight into a current topic of psycholinguistics topic – the mental lexicon; (3) the results of the present study are framed in the context of Husserl’s (1970, 1977) phenomenology, where human mental processes are considered as essential for the construction of the objective world. People create ideas about their existence *in* the world and belongingness *to* the world, which the free association task with the cue words *self* and *world* appropriately stimulates. In this way, the present study provides a particularized insight into the underlying structures, which constitute the inner meaning of world’s belongingness. Moreover, analysis of the category network shows that associations are mutually inter-related and that the concepts of *self* and *world* are internally connected via direct and mediated dependences, which reflects the structuring of perception and understanding of *self* and *world* in people’s minds.

It is also appropriate to consider the limitations of the present study regarding culture: the sample of Czech people, mostly university students, and also the fact that all of the acquired associations stem from the Czech language repertoire. On the other hand, the Czech Republic currently represents a unique mono-cultural non-religious laboratory in the middle of Western world (see e.g., Kuška et al., 2015), so the results of the free association task could be used as control-group data in comparison with another multicultural sample. The method of data collection, their categorization and subsequent analyses is universal.

## Author Contributions

MK is the principal author of this article and contributed to all of its sections. RT, the second author, is the author of the main parts of the Method and Discussion sections as well a co-author of the Results and the Conclusion. AAK designed and conducted the statistical operations and is the main author of the Data analysis section of the article, and partially of the Results section. JR is the author of the Introduction and Results sections. All four co-authors critically revised the entire text and worked together on the final version of the article.

## Conflict of Interest Statement

The authors declare that the research was conducted in the absence of any commercial or financial relationships that could be construed as a potential conflict of interest.
